# Supplementary material for: Issues Related to the Treatment of H. pylori Infection in People Living with HIV and Receiving Antiretrovirals
Source: Microorganisms. 2022 Jul 29;10(8):1541. doi: 10.3390/microorganisms10081541 (PMC9413132; doi:10.3390/microorganisms10081541)
Supplement: Supplementary file 1 [file microorganisms-10-01541-s001.zip › microorganisms-1794878-supplementary.pdf]

Table S1. Visual colored-based drug interactions between anti-*Helicobacter pylori* drugs and antiretrovirals.

Antiretro-virals	Pantoprazole	Lansoprazole	Esomeprazole	Omeprazole	Rabeprazole	Amoxicillin	Clarithromycin	Bismuth (sous citrate)	Tetracycline	Metronidazole	Ciprofloxacine	Levofloxacine	Rifabutin
Abacavir													
Didanosine													
Emtricitabine													
Lamivudine													
Stavudine													
Zidovudine													
Tenofovir													TDF
Efavirenz													
Etravirine													
Névirapine													
Doravirine													
Rilpivirine											!	!	
Atazanavir											!	!	
Darunavir													
Cobicistat													
Fosamprénavir							?						
Ritonavir													
Saquinavir													
Tipranavir											!	!	
Lopinavir													
Enfuvirtide													
Maraviroc													
Raltegravir											*		
Dolutegravir													
Elvitegravir													
Bictegravir													
Cabotegravir (oral)													
Fostemsavir													
Delavirdine													

Color legend

	Recommended
	Not recommended
	Caution, may be not recommended for *H. pylori* eradication
	Not studied, may be recommended
?	Clinical significance is uncertain, Monitor for adverse effects of both drugs
	Bismuth has not been studied, may be recommended
*	Poten
!	Risk of QT interval prolongation, an electrocardiogram is requested

TDF Tenofovir disoproxil Furamate

The online version of this table 1 is presented with comments for drug interactions
